# Antibody-Dependent Cellular Phagocytosis of HIV-1-Infected Cells Is Efficiently Triggered by IgA Targeting HIV-1 Envelope Subunit gp41

**DOI:** 10.3389/fimmu.2020.01141

**Published:** 2020-06-09

**Authors:** Maxence Duchemin, Daniela Tudor, Andréa Cottignies-Calamarte, Morgane Bomsel

**Affiliations:** ^1^Laboratory of Mucosal Entry of HIV-1 and Mucosal Immunity, Department of Infection, Immunity and Inflammation, Cochin Institute, CNRS UMR 8104, Paris, France; ^2^INSERM U1016, Paris, France; ^3^Université Paris, Paris, France

**Keywords:** IgA, HIV-1 envelope gp41, phagocytosis, ADCP, FcαRI/CD89, neutrophils, monocytes

## Abstract

Antibodies mediate a broad array of non-neutralizing Fc-mediated functions against HIV-1 including antibody-dependent cellular cytotoxicity (ADCC) and antibody-dependent cellular phagocytosis (ADCP). Accordingly, ADCC and ADCP induced by anti-HIV envelope gp120 IgG have been correlated to the limited success of the HIV-1 phase III vaccine trial RV144. It remains elusive whether ADCP can also be triggered by IgA, the isotype predominant at mucosal surfaces through which HIV-1 is mainly transmitted. Yet, we have previously shown that the HIV envelope subunit gp41-specific broadly neutralizing antibody 2F5 under the IgA isotype (2F5-IgA) triggers ADCC and cooperates with 2F5-IgG to increase HIV-1-infected cell lysis. Here, we now demonstrate that 2F5-IgA, more efficiently than 2F5-IgG, induces ADCP not only of gp41-coated beads but also of primary HIV-1-infected cells in a FcαRI-dependent manner. Both primary monocytes and neutrophils can act as effector cells of 2F5-IgA-mediated ADCP, although with different kinetics with faster neutrophil phagocytosis. However, unlike for ADCC, 2F5-IgA and 2F5-IgG do not cooperate to increase ADCP. Altogether, our results reveal that gp41-specific IgA mediate the efficient phagocytosis of HIV-1-infected cells. Inducing such ADCC and ADCP-prone IgA response by vaccination in addition to anti-HIV envelope IgG, might increase the protection against HIV acquisition at mucosal level.

## Introduction

By acting as a bridge between adaptive and innate immunity, antibodies (Abs) against Human Immunodeficiency Virus 1 (HIV-1) could participate to an increased protection against virus acquisition *in vivo*. Hence, extensive evidence from natural infection, preclinical studies conducted in non-human primates (NHP) (1), and vaccine trials such as the RV144 one, point to a critical role for antibody Fc receptor (FcR) engagement in reducing the risk of HIV-1 infection, decreasing post-infection viremia, and delaying viral rebound [reviewed in (2–4)].

Fc-mediated functions, including Antibody-Dependent Cellular Cytotoxicity (ADCC), Antibody-Dependent Cellular Phagocytosis (ADCP) and Antibody-Dependent Cell-mediated Viral Inhibition (ADCVI), are based on the recruitment of innate immune effectors expressing FcRs, such as monocytes/macrophages, neutrophils, dendritic cells and NK cells [reviewed in (5)], all able to eliminate the opsonized pathogen or infected cell.

Remarkably complex, Fc-mediated functions depend on antibody isotype, subclass, degree of glycosylation, body distribution, and effector cell pattern of FcR expression (6, 7). Moreover, the signals generated by a given FcR and consequently, the activities induced following Fc binding to its cognate FcR receptor depend on both the Ab isotype and the FcR subclass, with each FcR eliciting different signals (8). In humans, receptors for IgG include the inhibitory receptor, FcγRIIb, and five activating Fc-receptors, namely FcγRI, -RIIa, -RIIc, -RIIIa, and -RIIIb. FcγRI is a high affinity receptor, whereas the others exhibit low to medium affinity for the IgG Fc region (9). Like IgG, IgA, the second most abundant Ab class in the serum and the major one at mucosal level, interacts with cell surface-expressed FcR, the best known being the Fc-receptor alpha RI (FcαRI), also known as CD89 (10).

Whereas, only IgG in immune complexes, but not uncomplexed IgG can bind FcγRs, except FcγRI, and can stimulate FcγR-mediated functions (8), both blood monomeric and mucosal polymeric IgA bind to FcαRI in a similar manner (11), either as immune complex or not. Though IgA, similar to IgG binding to FcγRI (12), may have a better affinity for FcαRI when in immune complexes (13).

ADCC is the best characterized Fc-mediated function in the field of HIV-1, mainly for non-neutralizing but also neutralizing IgG [reviewed in (14)]. Likewise, we have previously demonstrated a correlation between the full protection of NHP following combined intramuscular and intranasal immunizations with a gp41 subunit-vaccine, and vaccine-elicited mucosal gp41-specific IgG triggering ADCC of HIV-1-infected cells (1). Furthermore, using as a model the HIV-1 envelope gp41-specific broadly neutralizing antibody (bNab) 2F5 under the IgA isotype (2F5-IgA), we recently demonstrated that IgA can trigger ADCC of HIV-1-infected primary CD4^+^T cells in a FcαRI dependent manner (15). Additionally, 2F5-IgA cooperates with 2F5-IgG, an antibody we previously demonstrated to trigger ADCC (16), and with 10E8-IgG, another gp41 bNab, in increasing target cell lysis (15).

Compared with ADCC, less is known about a possible protective role of ADCP in HIV-1 infection. The first line of evidence comes from studies showing a role of ADCP in protection against extracellular bacterial and fungal pathogens, and in clearing viral intracellular infections, including Influenza virus, West Nile Virus, adenovirus, SARS coronavirus, and foot-and-mouth disease virus [reviewed in (17)]. Concerning HIV-1, ADCP has been associated with a reduced risk of infection in NHP vaccine studies (18, 19). In RV144, ADCP was associated with the antibody responses that correlated with decreased risk of infection (20). In HVTN 505 ADCP correlated with decreased HIV-1 risk (21).

In HIV-1-infected individuals, the expression of the various FcγRs vary with disease progression, the high affinity FcγRI being increased in acute infection, whereas the activating receptor FcγRIIa, one other receptor responsible for IgG-mediated ADCP, as well as the FcγRIII, were reduced in chronically infected patients (22). Moreover, HIV-1 controllers and untreated slow progressors exhibited greater HIV-1 phagocytic activities compared with HIV-1-infected subjects under antiretroviral therapy. This enhanced phagocytic activity in absence of treatment is related to an altered IgG glycosylation linked to a more efficient binding to the activating FcγRIIa than the inhibitory FcγRIIb (7). An association between Fc-receptor genetics and disease progression or risk of infection was also evidenced in HIV-1-infected patients. Hence, patients homozygote for the low affinity allele of the FcγRIIa, which is involved in ADCP triggered by HIV-1-specific IgG, compared to those heterozygote for this allele have a significantly predicted accelerated rate of disease progression (23).

*In vivo*, ADCP was also associated with a reduced risk of infection in SIV (24) and SHIV (25) infection of NHPs, although the route of immunization, the type of effector phagocyte and the isotype, namely IgG or IgA, were associated with different ADCP outcomes (24). Following NHP immunization with a DNA/rAd5 vaccine, THP-1 monocytic cell-mediated ADCP of gp120-coated beads triggered by vaccine-induced IgG is associated with a reduced of risk of SIV infection when animals are vaccinated by the intramuscular route, while neutrophil phagocytosis triggered by vaccine-induced IgA is associated with a reduced risk of infection upon immunization by the nasal, but not the intramuscular route (24). Similarly, anti-gp120 IgG triggered ADCP of HIV-envelope gp120-coated beads mediated by monocytes and neutrophils correlate with protection and a reduced risk of infection by low dose SHIV challenges in a pentavalent HIV-1 vaccine study (25).

In addition to Fc-receptor genetics, ADCP is regulated by other factors such as the type of phagocyte, the combination of receptors expressed, the tissue immune environment as well as the antibody specificity, isotype, subclass, and glycoforms [reviewed in (26)]. Hence, a differential distribution of professional phagocytes was described among various mucosal tissues (27), with macrophages being the dominant phagocyte population in lymph nodes and intestinal tissues, whereas neutrophils represent the dominant phagocyte population in tissues from the lower female reproductive tract. When comparing the colon with the cervix, colon resident macrophages appear deficient in ADCP compared to colon- and cervix-resident neutrophils as well as cervix-resident macrophages (27). These results suggest that ADCP may play an important role in controlling or preventing HIV-1 infection and spread, although in a tissue differential manner.

Whereas, ADCP triggered by anti-HIV IgG has been studied, very limited information is available concerning the capacity of anti-HIV IgA to trigger ADCP. One of the rare studies on IgA-mediated phagocytosis reports that when opsonized by the CD4-binding site-specific CH31 bNab under the IgA1 or 2 isotype, HIV envelope-coated beads are phagocytosed by monocytes in an FcαRI dependent manner. However, in parallel experiments, opsonized virus ADCP is much less efficient (17). Furthermore, in breast milk and serum from HIV-infected mothers, IgA triggered ADCP of virions or HIV envelope-coated beads by monocytes is not usually detected (26). However, in none of these studies was HIV-infected cell phagocytosis evaluated.

Altogether, less evidence account for the role of the HIV-1 specific IgA in the protection mediated by the phagocytes at mucosal level, the main portal entry for HIV-1, where IgA antibodies play a major role in the protection. The specific engagement of FcαRI in ADCP of HIV-1, either as free virus or infected cells at mucosal level, is not yet defined, and could differ from that triggered by IgG.

Whereas, most FcR-mediated studies have evaluated antibodies to gp120, the most accessible HIV-1 envelope subunit but the most variable one, much less is known about those targeting gp41 that exposes accessible domains including the membrane proximal region (MPER) at the resting state (28), and is well-conserved. The role of mucosal gp41-specific IgA in the *in vivo* protection was best exemplified in highly exposed seronegative (HESN) individuals, this protection being mainly correlated with their neutralizing and transcytosis blocking activity (29–32). Whether mucosal anti-gp41 IgA from HESN (33) or those from fully protected monkeys following virosome-gp41 subunit vaccination (1) also mediate the phagocytosis of free virus or HIV-1-infected cells, following interaction of the Ab Fc-domain with its cognate FcR expressed on the innate effector cells was never studied.

Therefore, to evaluate the potential role of the gp41 specific IgA to trigger the phagocytosis of HIV-infected cells by ADCP, we took advantage of the 2F5-IgA we have recently shown to induce the lysis of infected target cells by ADCC (15). Moreover, we compared the efficacy of ADCP induced by 2F5-IgA with that of four HIV-1 IgG bNabs (i.e., 2F5-IgG, 4E10, 10E8, and 2G12). We finally evaluated whether gp41-specific IgA and IgG Abs together could synergize phagocytosis.

## Materials and Methods

### Antibodies and Peptides

2F5-IgA2 (2F5-IgA) and IgG1 (2F5-IgG) were obtained as previously described in Tudor et al. (34) and 2F5-IgA was purified using peptide M/Agarose purification (InvivoGen, San Diego, CAL, USA) following manufacturer's instructions. Irrelevant human IgA and IgG controls were from The Binding Site Group Ltd. (Birmingham, UK) and Jackson ImmunoResearch Europe Ltd. (Suffolk, CB8 7SY, UK), respectively. Biotinylated-P1 Peptide (aa 650–685) derived from gp41 envelope subunit of Clade B (P1) as described in Alfsen and Bomsel (35) was chemically synthesized by Eurogentec (Seraing, Belgium).

### HIV-1 Viruses

Viruses were obtained from the NIH AIDS Reagent Program: the HIV-1 JR-CSF (clade B, R5-tropic) expression plasmid was used for JR-CSF HIV-1 virus stock production by transfection of 293T cells as described in Tudor et al. (29).

### Target Cells

Target primary CD4^+^T lymphocytes were purified from healthy donor PBMCs by negative selection using human CD4^+^T enrichment kits (StemCell Technologies, France). For each experiment, a pool of PBMCs from three different healthy donors were activated with PHA for 48 h at 37°C. Then 3 × 10^6^ CD4^+^T were infected with 240ng p24 of JR-CSF clade B HIV for 2 h at 37°C and furthered cultured for 3–4 days. Infection was monitored by intracellular p24 [(Anti-Gag p24 KC57 clone conjugated to fluorescein isothiocyanate (FITC) (Beckman Coulter, Brea, CAL, USA)] detection using flow cytometry as described in Tudor et al. (29), resulting in 10–40% infected (p24^+^) viable cells.

The human CD4^+^T CEM-NK-resistant (CEM-NKr) lymphocytic cell line expressing CCR5 was obtained from the NIH AIDS Reagent Program. Infected CEM-NKR target cells were obtained by infecting 3 × 10^6^ CEM-NKr lymphocytes with 240ng p24 of JR-CSF, resulting in 30–40% p24^+^ viable cells. Infection was monitored by intracellular p24 detection as described in Tudor et al. (29).

### Effector Cells

Primary human monocytes and neutrophils were purified from healthy donor PBMC by negative selection using human monocyte enrichment kits (StemCell Technologies, France) and positive selection using MACSxpress® Human Whole Blood Neutrophil Isolation Kit according to manufacturer's protocol (Miltenyi Biotec, Bergisch Gladbach, Germany), respectively. Cells were carefully manipulated to avoid neutrophil activation. For each experiment, a pool of PBMCs from three different donors was used to purify monocytes and neutrophils to limit donor variability.

### Standard ADCP Assay Using HIV-1 Peptide-Coated Beads

The standard bead ADCP assay was adapted from Ackerman et al. (36). Briefly, 3 μl of stock 1 μm FITC FluoSpheres® NeutrAvidin®-labeled microspheres (ThermoFisher) were washed in PBS containing 2% BSA (PBS 2%BSA) and incubated with 0.2 μg of biotinylated P1 in 500 μl of PBS 2%BSA overnight at 4°C. Beads were washed in PBS 2%BSA to remove unbound P1, and resuspended in RPMI 1640 containing 10% FCS (R10) at a 1:100 dilution. 20 μl of P1-coated beads were incubated in triplicated wells with P1-specific Abs 2F5-IgA/2F5-IgG or gp120-specific 2G12-IgG at indicated concentrations in RPMI 1640 containing 10% FCS (R10) for 30 min at 37°C. Irrelevant human IgA2/IgG1 (hIgA/hIgG) at the same concentrations were used as a negative control. 4 × 10^4^ effector monocytes were then added to opsonized beads. Plates were spun 1 min at 300 g to promote contacts and further incubated for 3 h at 37°C. Cells were fixed in 4% paraformaldehyde and were immediately acquired on a Guava easyCyte flow cytometer (Merck-Millipore). Phagocytic score was determined by the percentage of cells with greater fluorescence than the 95th percentile of fluorescence from a no-antibody control, multiplied by their MFI as described in Tay et al. (37). To calculate P1-specific phagocytic score, phagocytic score obtained in the presence of hIgA/hIgG was deducted from phagocytic score obtained in the presence of HIV-1 specific antibody. Notably, hIgA and hIgG gave an unspecific phagocytic score of 223 and 288 for 0.1 μg/ml of hIgA and hIgG, respectively.

### ADCP-SHIP Assay of HIV-1 Peptide-Coated Beads

The bead ADCP-Specific Hybridization Internalization Probe (SHIP) assay protocol was adapted from Ana-Sosa-Batiz et al. (38). Briefly, 3 μl of stock 1 μm FITC FluoSpheres® NeutrAvidin®-labeled microspheres (ThermoFisher) were washed in PBS containing 2% BSA (PBS 2%BSA) and incubated with 0.2 μg of biotinylated P1 and 3 μl of 150 μM biotin- and Cy5-labeled fluorescent internalization probe (FIP_Cy5_) [(5′Cy5-TCAGTTCAGGACCCTCGGCT-Biotin 3′) (integrated DNA Technologies, Coralville, IA, USA)] in 500 μl of PBS 2%BSA overnight at 4°C. Beads were washed in PBS 2%BSA to remove unbound P1 and resuspended in R10 at a 1:100 dilution. 20μl of the prepared beads were incubated in triplicated wells with 2F5-IgA, 2F5-IgG, or 4E10-IgG at indicated concentrations or the irrelevant hIgA/hIgG in R10 for 30 min at 37°C. 4 × 10^4^ effector THP-1 or primary monocytes were then added. When mentioned, primary monocytes were pre-treated with 10 μg/ml of actin inhibitor cytochalasin D (Thermo Fisher Scientific, Waltham, MA, USA) for 30 min at 37°C. Plates were spun 1 min at 300 g to promote contacts and further incubated for 3 h at 37°C. Cells were then washed with ice cold PBS and surface-bound beads were quenched by adding 50 μg/ml of the complementary quenching probe (QP_C_) [(5′ -AGCCGAGGGTCCTGAACTGA-BHQ2- 3′) (Integrated DNA Technologies, Coralville, IA, USA)] for 15 min at 4°C. Cells were washed in PBS, fixed in 4% paraformaldehyde (PFA), and immediately acquired on a Guava easyCyte flow cytometer (Merck-Millipore). Flow cytometry allows distinguishing internalized beads (Cy5^+^) from surface bound beads (Cy5^−^). Thus, % of specific ADCP is calculated as follows: *% of FITC*^+^ *Cy5*^+^ *cells in the presence of antibody—background % of FITC*^+^ *Cy5*^+^ *cells without antibody*. To calculate P1-specific beads ADCP %, beads ADCP % obtained in the presence of hIgA/hIgG was deducted from beads ADCP % obtained in the presence of HIV-1 specific antibody. Notably, hIgA and hIgG gave an unspecific % beads ADCP of 1.7 and 2% respectively.

### ADCP of HIV-1-Infected T-Cells

3 × 10^4^ HIV-1-infected CD4^+^T target cells (TCs) were incubated in triplicated wells with either 2F5-IgA, 2F5-IgG, or 2G12-IgG at indicated concentrations or the hIgA/hIgG in R10 for 30 min at 37°C. Subsequently, primary monocytes or neutrophils used as effector cells (ECs) were stained with 0.1 μM intracellular CellTracker™ Deep Red Dye (DR) (Thermo Fisher Scientific, Waltham, MA, USA) in PBS for 20 min at 37°C, reaction was stopped by addition of 10 ml of R10 and cells were washed in R10. 9 × 10^4^ ECs were then added at a 1:3 TC:EC ratio to TCs and incubated at 37°C for 15 min to 3 h when using monocytes as effectors, or from 5 min to 18 h when using neutrophils as effectors. At the end of incubation, cells were washed, fixed with 4% PFA, permeabilized and stained with an anti-p24-FITC antibody (KC57, Beckman Coulter, France) for 25 min at RT. Finally, cells were washed and analyzed by flow cytometry using the gating strategy shown in Supplementary Figure 5. ADCP is determined by the percentage of DR^+^ effector cells with fluorescence > 5th percentile of p24-FITC fluorescence from a no-antibody control. Then, HIV-1 specific ADCP was calculated as follows: HIV-1 specific ADCP = *% DR*^+^ */ p24*^+^ *cells in the presence of antibody – % DR*^+^ */p24*^+^ *cells in the presence of hIgA/hIgG*. Notably, incubation with hIgA and hIgG gave an unspecific % of infected cells ADCP of 4 and 2.3% respectively.

### FcR Blockade

When indicated, monocytes effector cells were preincubated with blocking anti-human CD89 monoclonal Ab (clone MIP7c) (Abcam, France) or anti-human CD64 monoclonal Ab (clone 10.1) (BD Pharmingen, USA), each at 10 μg/ml, at 37°C for 1 h before addition of relevant ADCP-inducing Abs and peptide-coated beads. ADCP blockade is calculated as follows: ADCP inhibition (%) = *100 – 100 x (Phagocytic score in the presence of blocking anti-FcR / Phagocytic score without anti-FcR blocking)*.

### Statistical Analysis

Statistical significance was analyzed by the two-tailed Student's *t*-test using Prism 5 (GraphPad, San Diego, CA, USA) software.

## Results

### 2F5-IgA Mediates P1-Coated Beads Phagocytosis by Monocytes via FcαRI

We first evaluated ADCP using the classical assay based on the uptake of fluorescent beads (36) coated with a gp41 MPER-derived peptide, and using monocytes as effector cells. As gp41 subunit, we used the P1 peptide (aa 650–685) that we characterized earlier as an extended gp41-MPER peptide including the canonical 2F5 epitope (ELDKWA). P1 allows for HIV-1 binding to its mucosal receptor Galactosyl Ceramide and mediates HIV-1 transcytosis across epithelial cells (35) as well as uptake by dendritic cells (39). When used as a gp41-derived immunogen in a preclinical vaccine strategy in NHPs (1), P1 coupled to virosomes induced *in vivo* protection against SHIV challenges that correlates with P1-specific IgG capable of mediating ADCC (1).

**Figure 1:**
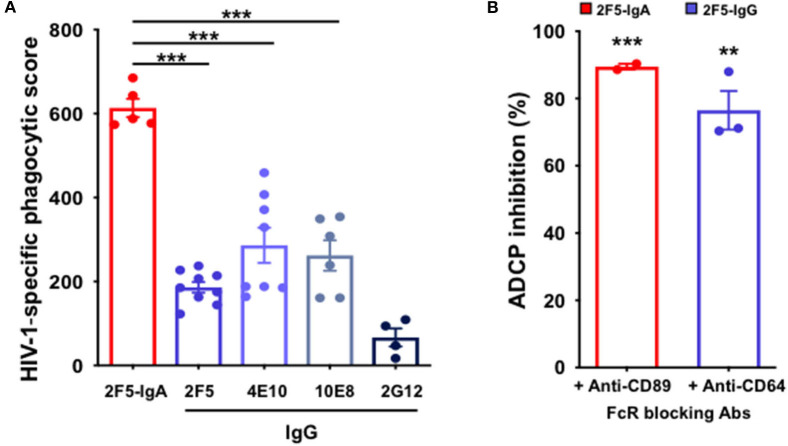
Anti-gp41 2F5-IgA and gp41-specific IgG induce ADCP of P1-coated beads in a FcαRI- and FcαRI-dependent manner. **(A)** 2F5-IgA (red bar) and gp41-specific IgGs (blue bars) mediate phagocytosis of P1-coated beads: Fluorescent beads were coated with P1, pre-incubated with the antibodies for 30 min at 37°C prior to addition to primary monocytes for phagocytosis for 3hrs at 37°C. gp120-specific 2G12-IgG (dark blue bar) or irrelevant human IgA2/IgG were used as negative controls. The P1-coated bead uptake was analyzed by flow cytometry and HIV-1 specific phagocytic score was calculated as indicated in the Method section. Values represent means of P1-specific phagocytic score obtained after the subtraction of the background phagocytosis levels induced by human IgA/hIgG ± SEM from 3 independent experiments performed in triplicate, ***p < 0.001, and, unpaired Student's *t*-test. **(B)** ADCP depends on FcαRI or FcγRI: Monocytes were pre-incubated with either anti-FcαRI (CD89) or anti-FcγRI (CD64) blocking antibody for 30 min at RT before addition to the immune complexes. Phagocytic score was calculated as in **(A)**. Values represent mean inhibition of phagocytic score in the presence of monocytes preincubated with anti-CD89 or anti-CD64 antibody ± SEM. **p < 0.01, ***p < 0.001 unpaired Student's *t*-test compared to ADCP in the absence of blocking antibodies.

We have previously shown that monocytes express both FcαRI/CD89 and FcγRI/CD64 and can therefore bind both IgA and IgG, respectively (15). Consequently, monocytes permit for a comparative evaluation of ADCP triggered by 2F5-IgA and the IgG bNabs, namely 2F5, 4E10, 10E8. Therefore, fluorescent P1-coated beads were opsonized with the various Abs prior to addition to primary blood monocytes used as effector cells for 3 h. Quantification of P1-coated bead uptake by flow cytometry shows that both 2F5-IgA and 2F5-IgG trigger ADCP, although with different efficacies. In all cases, 2F5-IgA is more efficient at triggering ADCP than its 2F5-IgG counterpart as well as other gp41-specific IgGs, namely 4E10 and 10E8. Accordingly, the phagocytic score calculated for 2F5-IgA at a 0.1 μg/ml concentration reaches 613 ± 23 but is significantly reduced for all gp41-specific IgGs, decreasing to 186 ± 13 for 2F5-IgG, 286 ± 42 for 4E10-IgG, and 262 ± 36 for 10E8-IgG. In contrast, ADCP induced by the anti-gp120 2G12-IgG, serving as negative control, remains negligible (Figure 1A).

Next, to evaluate the dependence of IgA-mediated phagocytosis on FcαRI, effector cells were preincubated with blocking anti-FcαRI/CD89 prior to initiating phagocytosis. Similarly, the engagement of FcγRI/CD64 in ADCP was evaluated by preincubation of monocytes with blocking anti-human CD64. Pre-incubation of monocytes with either anti-CD89 or with anti-CD64 results in a strong decrease in ADCP score, with a reduction of 89% ± 1 for 2F5-IgA and of 77% ± 6 for 2F5-IgG, compared with ADCP measured in the absence of FcR-blocking Abs (Figure 1B). These results indicate that FcαRI and FcγRI control IgA- and IgG-triggered ADCP, respectively.

### 2F5-IgA Triggers ADCP by Monocytes With Higher Efficacy Than Anti-gp41 IgGs, as Evaluated by the ADCP-SHIP Method

One pitfall of the classical ADCP assay described above is that it measures total beads associated with the effector cells, both surface-attached and intracellular (36), but cannot discriminate between strictly phagocytosed beads from that remaining exclusively attached to the effector cell surface, the later corresponding to 30% of the total cell-associated beads (17). Furthermore, the score used to quantify ADCP in this method does not discriminate the efficiency of ADCP (number of beads phagocytosed per effector cell) from the fraction of effector cell capable of phagocytosis. To overcome these limitations, we next evaluated ADCP using the recently developed ADCP-SHIP method (38) in which a fluorescent probe, namely the Cy5^+^ Specific Hybridization Internalization Probe (SHIP) (40) is coated on the FITC^+^ fluorescent beads, in addition to the HIV-envelope subunit P1. After 3 h of phagocytosis, a quencher of the SHIP fluorescence is added at 4°C to selectively inactivate the Cy5^+^ SHIP fluorescence of the beads attached at the effector cell surface but not that of the truly phagocytosed beads that are protected from the quencher due to their intracellular localization (38). Evaluation of fluorescent cells by flow cytometry directly quantifies the % of cells that have internalized beads corresponding to double-positive FITC^+^Cy5^+^ monocytes, discriminating the FITC fluorescence corresponding to surface-bound beads and the FITC^+^ single positive monocytes covered by surface-bound beads.

**Figure 2:**
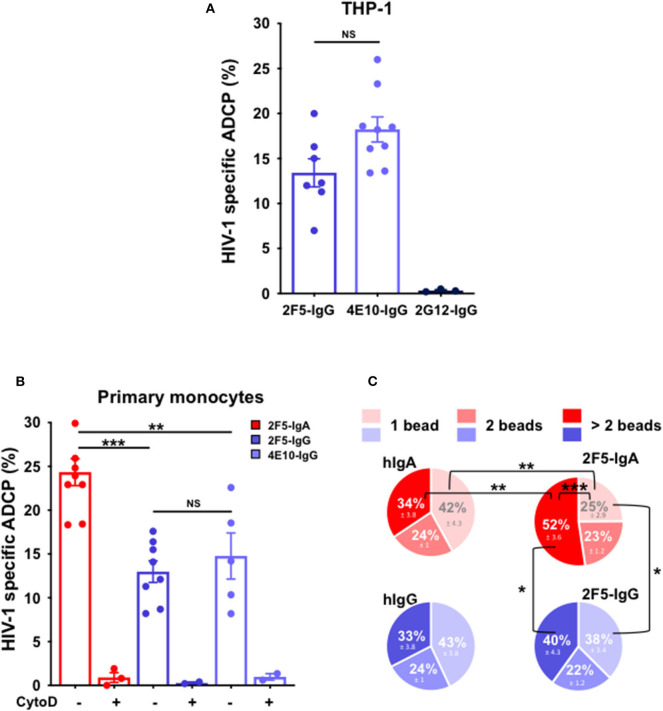
Anti-gp41 2F5-IgA triggers more efficient ADCP of P1-coated beads compared to anti-gp41 2F5-IgG and 4E10-IgG in the ADCP-SHIP assay. Genuine ADCP of beads triggered by gp41-specific IgA and IgG was evaluated by the ADCP-SHIP method, discriminating surface-attached beads from actual intracellular phagocytosed beads. **(A)** ADCP-SHIP method validated using the monocytic THP-1 as effector cells. Fluorescent neutravidin beads coated with Cy5-labeled fluorescent internalization probe (FIP_Cy5_) and gp41 subunit P1 were incubated with anti-gp41 2F5-IgG (blue bar), and 4E10-IgG (light blue bar), anti-gp120 2G12-IgG (dark blue bar) or irrelevant human IgA2 or IgG prior to addition to the THP-1 effector cells for 3 h at 37°C. Cy5 fluorescence of surface-bound beads was quenched by incubation with the complementary quenching probe (QP_C_). ADCP was evaluated by flow cytometry and calculated as follows: % of FITC^+^ Cy5^+^ cells with antibody—background % of FITC^+^ Cy5^+^ cells without antibody. ADCP % in the presence of control human IgA/hIgG was subtracted from ADCP % triggered by HIV-1 specific antibody. Values represent means of P1-specific beads ADCP % ± SEM from 3 independent experiments performed in triplicate. NS: *p* > 0.05, unpaired Student's *t*-test. **(B)** Primary monocyte-mediated ADCP is more efficiently triggered by 2F5-IgA than gp41-IgGs. 2F5-IgA (red bar), 2F5-IgG (blue bar) and 4E10-IgG (light blue bar) were assayed by the ADCP-SHIP method using primary monocytes as effector cells. When indicated, monocytes were pre-treated with 10 μg/ml of Cytochalasin D (CytoD) for 30 min at 37°C or left untreated and added to beads opsonized with the IgA/IgG Abs. Values represent means of P1-specific beads ADCP % ± SEM from at least 3 independent experiments performed in triplicate, NS *p* > 0.05, **p < 0.01, ***p < 0.001, unpaired Student's *t*-test. **(C)** Characterization of the efficiency of bead phagocytosis by ADCP-SHIP. The number of beads phagocytosed by each cell triggered by 2F5-IgA/IgG or the control hIgA/hIgG was evaluated from the intensity of Cy5 fluorescence associated to each cell. Light, median, or dark red/blue represent the percentage of cells having phagocytosed 1, 2, or >2 beads (i.e., between 3 and 7). Values represent means of P1-specific ADCP distribution % ± SEM, from 3 independent experiments performed in triplicate, *p < 0.05, **p < 0.01, ***p < 0.001, unpaired Student's *t*-test.

To validate the ADCP-SHIP assay, we first evaluated ADCP activity of two anti-gp41 bNAbs, namely 2F5 and 4E10, using the monocytic THP-1 as effector cells that express all FcγR (16, 17). As shown in Figure 2A, both 2F5 and 4E10 IgGs trigger phagocytosis of gp41 subunit P1-coated beads with a similar efficacy of 13.4% ± 1.6 for 2F5 and 18.2% ± 1.4 for 4E10 (student's *t*-test *p* = non-significant), respectively, but not the anti-gp120 2G12-IgG used as negative control (Figure 2A).

Next, as THP-1 lacks FcαRI expression [(17) and not shown], blood monocytes were used as effector cells in the ADCP-SHIP assay to evaluate P1-coated bead ADCP triggered by 2F5-IgA. As a result, 2F5-IgA induces ADCP more efficiently than its 2F5-IgG counterpart, but also than the 4E10-IgG (Figure 2B), with a respective efficacy of 24.4% ± 1.5 compared with 13% ± 1.2 and 14.8% ± 2.6, respectively (student's *t*-test *p* < 0.005 comparing the IgA with either IgG antibody). Moreover, this ADCP is specific as pre-treatment of monocytes with the actin inhibitor cytochalasin D (CytoD), frequently used to inhibit phagocytosis and one hallmark of phagocytosis (41), impairs bead internalization (Figure 2B).

To evaluate the phagocytosis efficacy at the single effector cell level, the number of beads phagocytosed by each effector cell was next quantified (Figure 2C), a specific feature made possible by the ADCP-SHIP assay rational. Thus, when triggered by 2F5-IgA, the majority of effector monocytes had phagocytosed more than 2 beads (52% ± 3.6), whereas only 25% ± 3.6 had phagocytosed only one bead (student's *t-*test *p* < 0.001). In contrast, when triggered by 2F5-IgG, an equal proportion of cells phagocytosed 1 and more than 2 beads (student's *t-*test *p* > 0.05), and monocytes having internalized more than 2 beads is thus decreased as compared with 2F5-IgA ADCP (40% ± 4.3, student's *t-*test *p* < 0.02) suggesting that the mechanism of phagocytosis might differ between the two isotypes in this model. Monocytes being professional phagocytes are known to phagocytose in the absence of relevant antibody. This phagocytosis by default, when evaluated in the presence of irrelevant human IgA (hIgA) or IgG (hIgG), results in a similar profile of phagocytosed bead number for both irrelevant antibodies, as expected, with a majority of monocytes having internalized only 1 bead compared to >2 beads. Furthermore, the mechanism of ADCP vs. non-specific phagocytosis appears different, at least for 2F5-IgA vs. hIgA, as the profile of phagocytosed beads differs when triggered by 2F5-IgA vs. non-specific hIgA. Indeed, specific 2F5-IgA triggered ADCP compared with irrelevant hIgA phagocytosis by default results in statistically significant higher percentage of cells that internalized >2 beads at the detriment of 1 bead exactly (Figure 2C). No difference is observed for cells having internalized 2 beads by the two mechanisms, most likely corresponding to residual phagocytosis. For 2F5-IgG triggered ADCP vs. by default phagocytosis in the presence of hIgG, a similar tendency in the phagocytosed intracellular bead profile is observed although not in statistically significant manner.

Altogether, these results show that 2F5-IgA induces the specific phagocytosis of the P1-coated beads by monocytes via FcαRI with a higher ADCP efficacy than other anti-gp41 bNabs acting via FcγRI.

### 2F5-IgA Triggers ADCP of HIV-1-Infected CD4^+^T Target Cells by Monocytes

The initial cells infected by HIV-1 upon transmission, and those that either replicate the virus in untreated patients or establish viral reservoirs represent one of the major obstacles in HIV-1 treatment and cure. Therefore, we next explored whether HIV-infected cells, whose size and HIV envelope antigen exposure diverge from that of beads, could be phagocytosed by ADCP triggered by 2F5-IgA as a potential elimination mechanism.

To date, in contrast to the numerous HIV-1 ADCC assays measuring infected cells lysis (42), and those measuring ADCP of HIV-envelope-coated beads (36), no model evaluating ADCP of HIV-1-infected target cells has been reported, yet. Therefore, the establishment of a reliable experimental model is needed to evaluate ADCP of pathogenesis-relevant target cells. First, we used as targets the CD4^+^T CEM cell line that are resistant to NK cells (CEM-NKr) infected with HIV-1 (JR-CSF) as target, which infection level reaches 30–40% of the total cell population after 3 days, as we have shown (15). Primary monocytes, expressing both Fcα- and Fcγ-R, pre-stained with the intracellular CellTracker™ Deep Red Dye were used as effector cells. Targets opsonized with 2F5-IgA, 2F5-IgG, or 2G12-IgG were incubated with monocytes for 3 h. Cells were then fixed, stained intracellularly with Gag p24-FITC, and analyzed by flow cytometry as indicated in the Method section. No phagocytosis of HIV-1-infected CEM target cells could be measured (Supplementary Figure 1), most likely for geometrical reasons because the CEM cell line is too large in size to be phagocytosed by the smaller primary monocytes.

**Figure 3:**
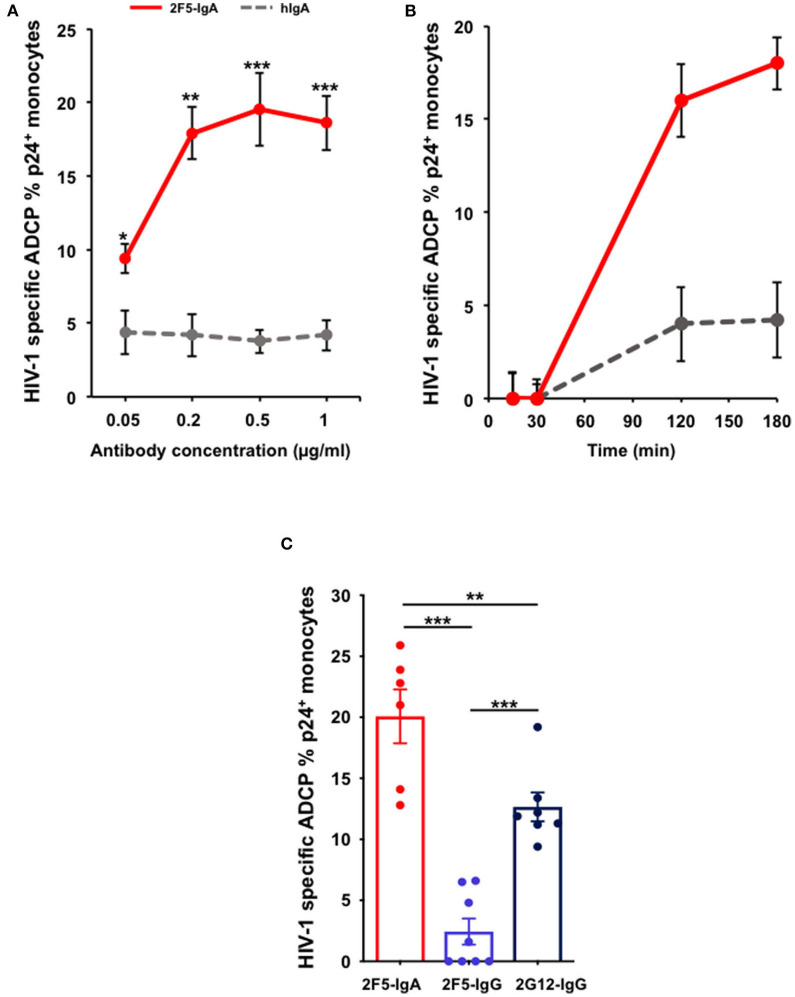
2F5-IgA mediates ADCP of HIV-1-infected primary CD4^+^T lymphocytes by monocytes more efficiently than 2F5-IgG and 2G12-IgG. Primary CD4^+^T-cells were infected with JR-CSF clade B HIV-1 for 72 h and incubated with **(A)** various concentrations of 2F5-IgA or hIgA2, **(B)** 0.2 μg/ml, or **(C)** 0.5 μg/ml of 2F5-IgA, 2F5-IgG, 2G12-IgG, or hIgA/IgG for 30 min at 37°C. Primary monocytes were stained with 0.1 μM intracellular CellTracker™ Deep Red Dye, added to opsonized infected target cells and allowed to phagocyte for 3 h at 37°C. Cells were washed, fixed in PFA, permeabilized before intracellular staining with anti-p24 FITC-coupled Ab and analyzed by flow cytometry. ADCP percentage is determined as indicated in the Method section. In **(A,B)**, non-specific ADCP mediated by hIgA2 are shown. In **(C)**, background ADCP % obtained in the presence of hIgA/hIgG was subtracted from ADCP % triggered by HIV-1 specific antibody. Values represent means of HIV-1-specific infected cells ADCP % ± SEM, from 4 independent experiments performed in triplicate, NS *p* > 0.05, *p < 0.05, **p < 0.01, ***p < 0.001, unpaired Student's *t*-test.

Therefore, to evaluate ADCP of HIV-1 infected cell, we replaced the cell line by primary blood CD4^+^T cells that are smaller than the monocytes and reach an infection range of 10–40% of the total target cell population upon *ex vivo* infection with HIV-1. In this case, applying the same protocol as above (Supplementary Figure 1), HIV-infected CD4^+^T lymphocytes opsonized by 2F5-IgA are efficiently phagocytosed by monocytes in a 2F5-IgA concentration-dependent manner, from 9.4% ± 1 when triggered by 0.05 μg/ml up to 18.6% ± 1.6 for 1 μg/ml of 2F5-IgA (Figure 3A). ADCP reaches a plateau at Ab concentration ≥ 0.5 μg/ml, suggesting that 2F5-IgA binding to target cells was saturated from this concentration or reached the prozone effect commonly observed in these types of assays (Figure 3A). When quantified kinetically from 15 min up to 3 h, specific 2F5-IgA mediated HIV-1 infected cell phagocytosis was detected only from after 2 h of effector and opsonized target cell incubation (Figure 3B). To detect eventual T-cell targets remaining attached to the monocyte surface, an additional CD3 staining was performed at the end of the phagocytosis period and prior to Gag p24 intracellular detection, which requires permeabilization. In this case, no CD3 signal associated with p24^+^ monocytes was detected. This indicated that in this assay, the p24^+^ monocytes we quantified correspond to intracellular HIV-infected CD4^+^T cells truly phagocytosed by monocytes and not attached to monocytes cell surface forming conjugates. Irrelevant hIgA used as negative control induced negligible ADCP target cell uptake at all concentrations tested (<5%) (Figure 3A).

Of note, although using a pool of effectors pooled from three donors could have induced a non-specific ADCP, this is not the case for the ADCP mediated by the antibodies we report here as at all concentrations tested, irrelevant human IgA induced negligible ADCP. 2F5-IgA triggered ADCP of HIV-infected cells is more efficient than that of 2F5-IgG, as observed for gp41 peptide-coated beads, and also statistically superior than the anti-gp120 2G12-IgG (Figure 3C). The difference between the percentage of monocytes that phagocytosed infected cells after FcR activation by 2F5-IgA or 2F5-IgG is probably due to the gp41 epitope exposure at the HIV-1-infected cell surface, more accessible for 2F5-IgA as compared to 2F5-IgG, as we previously shown (15), and less to the stoichiometry of FcR binding (43), since 2G12-IgG opsonized HIV-infected cells are efficiently phagocytosed by monocytes (Figure 3C). Furthermore, apoptotic cells that are present in the population of HIV-1-infected cells do not influence phagocytosis mediated by the antibodies, as no difference of ADCP mediated by 2F5-IgA and -IgG was observed after dead cell removal (Supplementary Figure 2).

### 2F5-IgA Triggers the ADCP of HIV-1-Infected CD4^+^T Cells by Neutrophils

Neutrophils are professional phagocytes that comprise more than 50% of leukocytes and are abundant at the mucosal level. As neutrophils express the phagocytosis-prone FcαRI/CD89, FcγRII/CD32, although not FcγRI/CD64 (not shown), we also evaluated their ADCP activity. Therefore, using the same strategy as above (Figure 3) applied to monocytes, neutrophils were stained with CellTracker™ Deep Red Dye prior to incubation with the opsonized HIV-infected CD4^+^T cells. However, in this case, ADCP was quantified from earlier time points, namely 5 min, and until 18 h.

**Figure 4:**
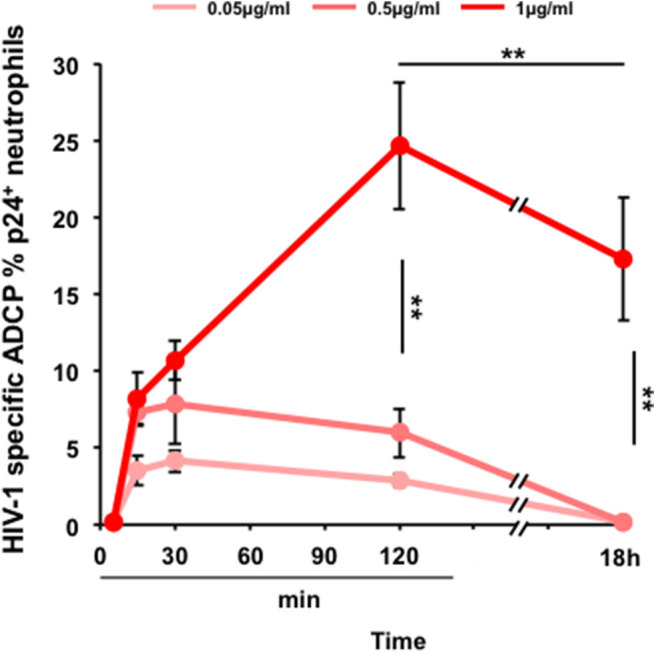
2F5-IgA-mediated ADCP of HIV-1-infected primary CD4^+^T lymphocytes by neutrophils. Primary CD4^+^T cells were infected with JR-CSF clade B HIV-1 for 72 h and incubated with various concentrations of 2F5-IgA or hIgA2 for 30 min at 37°C. Primary neutrophils were pre-stained with 0.1 μM intracellular CellTracker™ Deep Red Dye, added to opsonized infected target cells and allowed to phagocytose for indicated times. Cells were washed, fixed, permeabilized, stained intracellularly with anti-p24 FITC Ab and analyzed by flow cytometry. ADCP percentage in each condition is determined as indicated in the Method section. Values represent means of HIV-1-specific infected cells ADCP % ± SEM, from 3 independent experiments performed in triplicate, **p < 0.01, unpaired Student's *t*-test.

As a result, neutrophils are capable of ADCP of HIV-infected cells triggered by 2F5-IgA in a concentration-dependent manner from 0.05 μg to 1 μg/ml (Figure 4). ADCP by neutrophils is detected already after 15 min with an efficacy of 3.5% ± 0.9, 7.3% ± 0.9 and 8.2% ± 1.7 for each concentration, respectively. ADCP reaches already a plateau at 15 min for a 2F5-IgA concentration of 0.05 and 0.5 μg/ml but for 2F5-IgA at 1 μg/ml, ADCP increases until 2 h. After 18 h of incubation, a high background of non-specific phagocytosis observed in the presence of hIgA used as negative control was detected (not shown), in turn reducing HIV-1 envelope-mediated ADCP to zero when triggered up to 0.5 μg/ml 2F5-IgA and to 17.3% ± 4 when triggered by 1 μg/ml of 2F5-IgA (Figure 4). However, unexpectedly, no phagocytosis of HIV-1-infected cells triggered by IgG, either anti-gp41 2F5 or -gp120 2G12, was observed for neutrophils (Supplementary Figure 4). Though in these cells, such IgGs have been shown to trigger ADCP of gp41 or gp120 subunit-coated beads (26) and in monocytes, such IgGs were prone, although in a limited manner, to ADCP of HIV-1-infected cells (Figure 3). These differences suggest that in addition to the determinant role of the isotype, namely IgA or IgG, in triggering ADCP as shown in this study, the mechanism of IgG/FcγR-mediated ADCP differs according to cell type and size of the target. In monocytes, FcγRI appears to mediate both small peptide-coated beads and viruses, and larger HIV-1-infected CD4^+^T lymphocytes targets whereas in neutrophils lacking FcγRI, the low affinity FcγRII/CD32 would only phagocytose coated beads (26), but not larger HIV-1-infected CD4^+^T lymphocytes targets.

Altogether, the present results indicate that 2F5-IgA can trigger ADCP of P1-coated beads by activating monocytes, but also for the first time, of HIV-1-infected target cells by both, monocytes and neutrophils, two types of effector cells that are present in the blood and at the mucosal level and thus relevant for HIV-1 pathogenesis.

## Discussion

Beyond neutralization breadth and strength, the antibody-dependent cellular cytotoxicity (ADCC) and antibody-mediated cellular phagocytosis (ADCP) have emerged as two important Fc-mediated anti-viral functions that may operate in the blood but also at the HIV-1 mucosal portal of entry where HIV-1 antibodies can activate the innate immune cells almost all expressing FcRs.

In addition to ADCC triggered by the MPER gp41-specific IgA (2F5-IgA) we previously described (15), we now assessed the capacity of 2F5-IgA to trigger ADCP by monocytes and neutrophils, two phagocyte effectors active in the blood and mucosa. Using various experimental models, we demonstrate that 2F5-IgA induces FcαRI-dependent phagocytosis of not only gp41 MPER-derived P1 peptide-coated beads that mimic virions, but also that of HIV-1-infected CD4^+^T lymphocytes, target cells critical in early HIV-1 transmission events and in the seeding of HIV-1 reservoirs. Whereas, primarily neutralization and FcγR-mediated functions driven by anti-HIV-1 envelope gp120 IgG have been mostly studied in the literature (42), our results altogether provide chief complementary insights on an additional HIV-1-protective function mediated by HIV-1 envelope gp41-specific IgA dependent on FcαRI signalization, in addition to the previous anti-viral functions such as neutralization, transcytosis blocking and ADCC that we already demonstrated (1, 15, 34).

Additionally, we evaluated the FcγR-dependent phagocytosis of three IgG bNAbs, two of them, 2F5 and 4E10, overlapping the conserved gp41 epitope of 2F5-IgA, the third, 2G12, binding to conserved gp120 N-linked glycans. Two main reasons support this comparative evaluation: (i) the capacity of the Fc region to modulate antibody paratope-mediated functions including antibody specificity, affinity and neutralizing activity as we recently demonstrated comparing 2F5-IgA with 2F5-IgG (34), (ii) the dependence of the signals generated by a given FcR, α or γ on both the Ab isotype and the FcR subclass (8), resulting in distinct Fc-mediated function efficacy such as ADCC, even for IgA and IgG with the same paratope, as we demonstrated (15).

Our results indicate that 2F5-IgA triggers ADCP more efficiently than anti-gp41 or -gp120 IgG bNAbs, irrespective of both the effector type, namely monocyte and neutrophils, and the target type, namely coated beads or HIV-1-infected cells. Several parameters controlling Ab-mediated functions could account for differences of IgG vs. IgA in triggering ADCP, including the antigen recognition by the paratope, and the isotype-specific Fc interaction with FcR.

Concerning the role of the paratope, IgA and IgG isotypes bind differentially to their cognate epitope, modifying in turn Ab affinity (34). In particular, 2F5-IgA and 2F5-IgG recognize different tri-dimensional regions on gp41 in addition to the canonical 2F5 epitope ELDKWA, resulting in a 50 fold higher affinity of the IgA over the IgG 2F5-isotype that translates into an increased ADCC (15), and ADCP of HIV-1-infected CD4^+^T cells, as we report here. Accordingly, 2G12-IgG and 2F5-IgA having an affinity for HIV envelope in the same range (44) trigger ADCP with similar efficacy. The epitope recognized by 2G12, specific for the mannose region and located at the apex of the trimer of gp120, could be more accessible than the MPER conformational 3D-epitope recognized by 2F5-IgG (34).

Concerning the role of Fc binding to its FcR, the stoichiometry of Ab/FcR binding differs between IgA and IgG. Indeed, an IgA molecule, even monomeric, binds to two FcαRI molecules whereas an IgG molecule binds only to one FcγR molecule (45). IgA binding to FcαRI is also rapidly enhanced by cytokine induced inside-out signaling, and can in turn enhance FcαRI valency and contribute to stronger avidity for IgA immune complexes (46), thus increasing ADCP efficacy compared to IgG. Furthermore, in a model of opsonized cancer cell killing by neutrophils, IgA-mediated binding to neutrophils is more stable compared to that of IgG, resulting in an IgA engagement of neutrophils capable of eliciting a stronger Fc receptor signaling than IgG, and in turn higher killing (43). Whether the higher ADCC (15) and ADCP we observed for 2F5-IgA compared to anti-gp41 IgG directly translate into a stronger signalization cascade downstream FcαR than FcγR stimulation remains however to be formally proven.

An additional parameter affecting ADCP is the type of the effector phagocyte in which the magnitude of the FcR signaling cascades induced by IgA or IgG differ (43). Accordingly, in neutrophils, FcαRI activation by 2F5-IgA results in a faster phagocytosis than that in monocytes, ADCP being initiated from 15 min and 2 h after contact with the target cell, respectively as shown here. Such fast IgA-triggered ADCP by neutrophils is in agreement with their rapid ability to eliminate bacteria (47), whereas bacterial killing by monocyte is less efficient/rapid (48). Additionally, IgG triggered ADCP of gp120-coated beads is faster in neutrophils than monocytes (49).

IgA and IgG are both present in body fluids and thus should be able to interact in their functions *in vivo*, as illustrated by the cooperation of anti-gp41 IgG with IgA in increasing ADCC (15). However, anti-gp41 IgG do not cooperate with IgA to increase ADCP of HIV-1-infected cells mediated by either monocytes (Supplementary Figure 3) or neutrophils (Supplementary Figure 4). Such lack of cooperation between the two isotypes might be ascribed to the cellular mechanism of ADCP that fully differs from that of ADCC targeting HIV-1-infected cells. Indeed, in ADCC, one effector cell can contact several opsonized targets, each by one or the two Ab isotypes, in turn triggering their lysis. In contrast in ADCP, following these multiple contacts with the IgA and/or IgG-opsonized target cells, the effector in turn engulfs the opsonized target, which, for geometrical reason, cannot be more than one per effector cell (50). Accordingly, we could not observe the phagocytosis of CEM-NKR cells which size is much larger than the monocyte one (Supplementary Figure 1). A steric hindrance resulting from opsonization by 2F5-IgA and 2F5-IgG may apply for other anti-gp41 IgG, as shown between other HIV-1 envelope antibodies (51).

Despite the various antiviral functions described (1, 29, 30, 32, 52–55), the role of IgA in protecting against HIV-1 remains controversial (56–58). Such differences between studies could be explained by the source of the IgA sampled in HIV-1-infected patients, vaccinees, or HESN resistant individuals, the sampling compartment, namely mucosal vs. systemic, and/or the HIV-1 envelope subunit targeted, namely gp120 vs. gp41. In addition, the experimental model used to assess Fc-mediated functions is also determinant. For instance, in the RV144 trial, the systemic anti-gp120 IgA response was associated with a decrease in vaccine-induced protection (56), suggesting a rather harmful role of IgA antibodies. Accordingly, vaccine-induced anti-gp120 IgAs have been proposed to compete with anti-gp120 IgGs, thereby reducing IgG-mediated ADCC (57). However, ADCC effector cells used in this later study lacked FcαRI/CD89 expression and therefore, the intrinsic Fcα-dependent ADCC activities of IgA and a potential synergy of the two isotypes could not be evaluated. Furthermore, extensive analyses of the rich set of data collected from RV144 vaccine recipients to identify associations between the Ab isotype and their Fc-mediated effector functions suggest that the induced IgA response is rather a marker for a deregulated and less functional immune response (59, 60). In addition, both high and low levels of induced IgA are found in patients who seroconverted during the trial, suggesting a lack of correlation with decreased vaccine efficacy.

However recently, two anti-gp120 monoclonal IgAs derived from B-lymphocytes of patients vaccinated were shown to trigger ADCP and to inhibit HIV-1 binding to Galactosyl Ceramide, the HIV-1 receptor on epithelial and dendritic cells allowing HIV-1 transcytosis (35) and uptake by dendritic cells (39), suggesting that these two IgA have protective functions at the mucosal level (61). The capacity of anti-gp41 IgA to trigger FcαRI-mediated ADCP by two types of phagocytes, as we report here, suggests that vaccine induced IgA might also have contributed to full protection against repeated mucosal viral challenges following vaccination with P1/gp41 virosomes, in addition to the FcγR-mediated ADCC triggered by anti-MPER specific IgG we had reported (1). Focusing on the humoral immunity against HIV-1 by analyzing the role of Fc-related responses for both IgA and IgG, and deciphering their anti-viral functions in addition to neutralization may initiate novel vaccination approaches.

Several parameters may limit our study. First, we used the 2F5 broadly neutralizing Ab expressed as IgA or IgG to evaluate ADCP, comparatively. 2F5-IgG has been shown to be polyspecific by binding the cytosolic enzyme kynureninase *in vitro* (62). However, this polyspecificity cannot account for 2F5 mediated ADCP as binding of 2F5-IgA and 2F5-IgG to non-infected cells was negligible (15, 34) and similar to the binding of irrelevant human IgG and IgA used as negative controls. Second, all forms of IgA have been shown to bind CD89 with similar affinities (11) and may thus have the potential of mediating ADCP. Although our study only evaluates the ADCP mediated by monomeric IgA, ADCP mediated by dimeric IgA, present in the mucosal tissue would be necessary to perform in order to fully validate the use of 2F5-IgA in a therapeutic strategy against mucosal HIV transmission.

Altogether, our present results provide new insights about the optimal *in vivo* activity profiles of gp41-specific IgA, as well as other anti-HIV-1 broadly neutralizing IgG that operate via Fc-mediated effector functions, and highlight that the efficacy of HIV-infected cells phagocytosis depends on antibody specificity and isotype. At mucosal level, the phagocytosis of either free virus or HIV-infected cells could limit the virus replication and thus establishment of the founder population responsible for the infection spread at the systemic level. Our new ADCP model targeting HIV-infected cells relevant for the AIDS pathology should provide a useful method, that was lacking until now, to address the phagocytosis functions of IgA but also IgG in future clinical trials.

## Data Availability Statement

The datasets generated for this study are available on request to the corresponding author.

## Author Contributions

MD, DT, and MB conceived and designed the experiments and wrote the paper. MD, AC-C, and DT performed the experiments. MD, AC-C, DT, and MB analyzed the data.

## Conflict of Interest

The authors declare that the research was conducted in the absence of any commercial or financial relationships that could be construed as a potential conflict of interest.
