# Supplementary figures and images for: Antibody-Dependent Cellular Phagocytosis of HIV-1-Infected Cells Is Efficiently Triggered by IgA Targeting HIV-1 Envelope Subunit gp41
Source: Front Immunol. 2020 Jun 9;11:1141. doi: 10.3389/fimmu.2020.01141 (PMC7296124; doi:10.3389/fimmu.2020.01141)

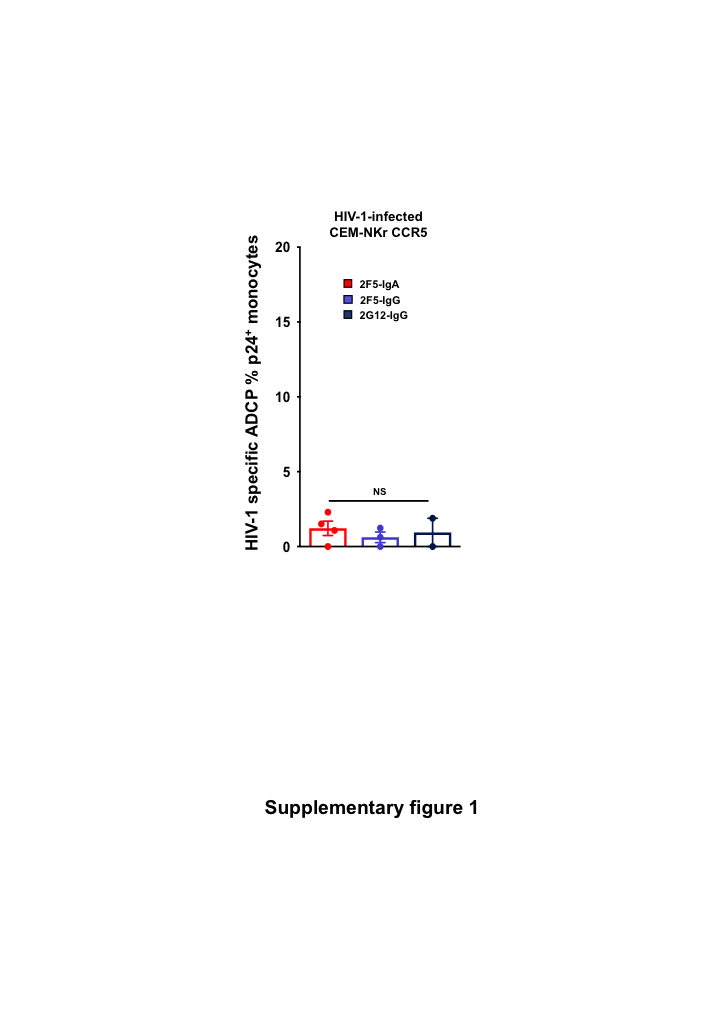

Supplement: Supplementary file 1 [file Image_1.TIFF]

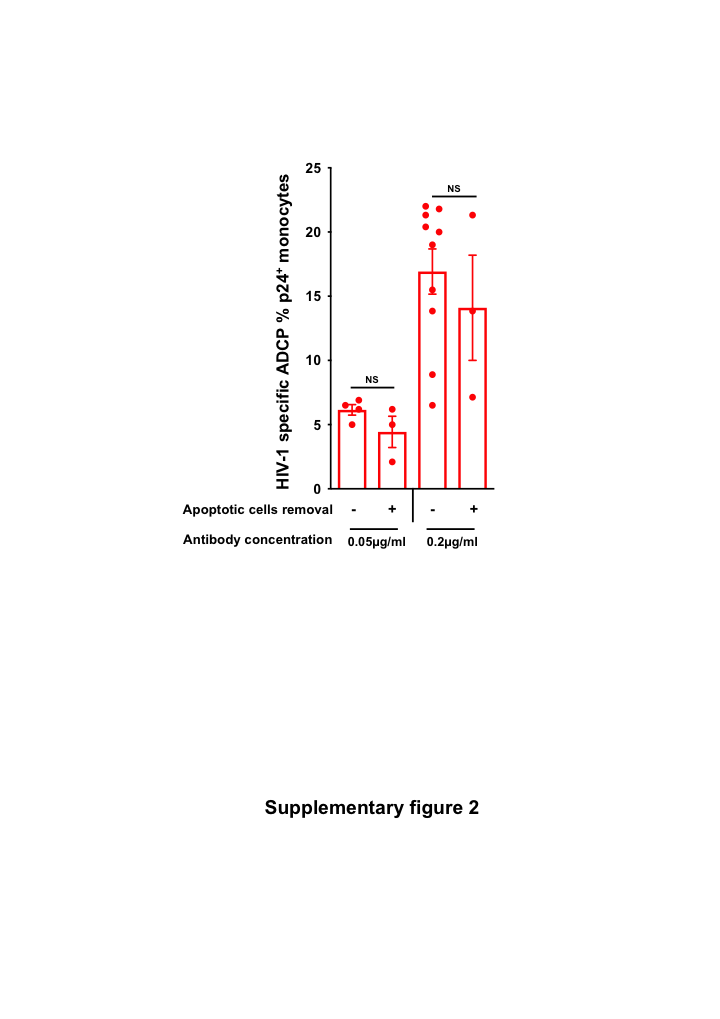

Supplement: Supplementary file 2 [file Image_2.TIFF]

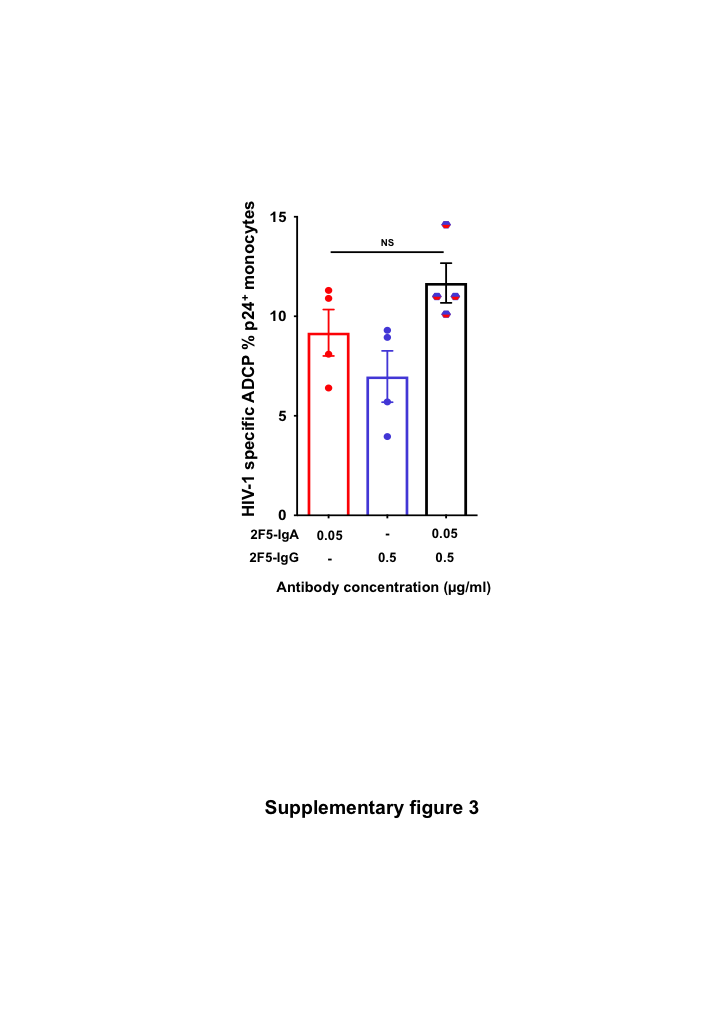

Supplement: Supplementary file 3 [file Image_3.TIFF]

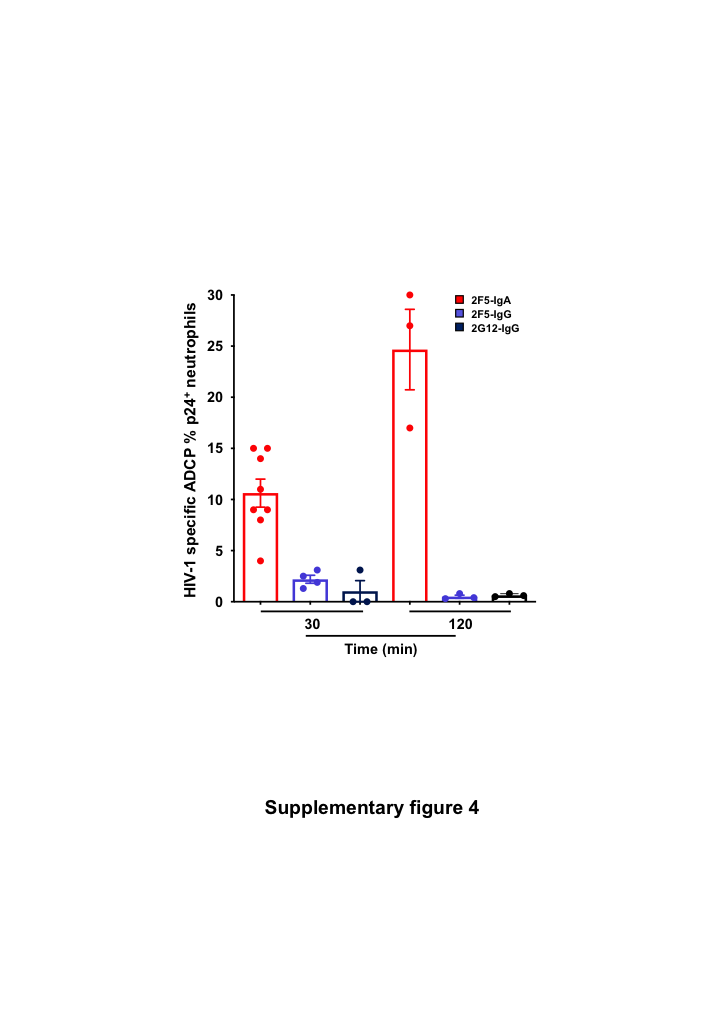

Supplement: Supplementary file 4 [file Image_4.TIFF]

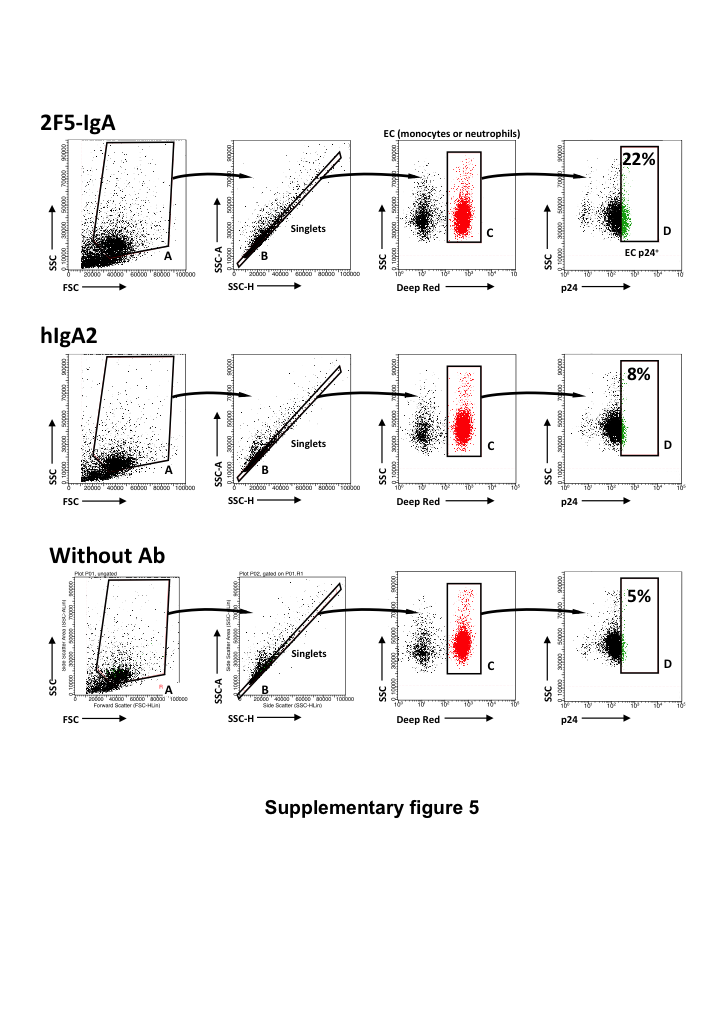

Supplement: Supplementary file 5 [file Image_5.TIFF]
