# Supplementary material for: Antibody-Dependent Cellular Phagocytosis of HIV-1-Infected Cells Is Efficiently Triggered by IgA Targeting HIV-1 Envelope Subunit gp41
Source: Front Immunol. 2020 Jun 9;11:1141. doi: 10.3389/fimmu.2020.01141 (PMC7296124; doi:10.3389/fimmu.2020.01141)
Supplement: Supplementary file 6 [file Presentation_1.pdf]

Antibody- Dependent Cellular Phagocytosis of HIV-1-infected cells is efficiently triggered by IgA targeting HIV-1 envelope subunit gp41

Maxence Duchemin[1,2,3], Daniela Tudor[1,2,3], Andréa Cottignies-Calamarte[1,2,3] and Morgane Bomsel[1,2,3]*

[1]Laboratory of Mucosal Entry of HIV-1 and Mucosal Immunity, Department of Infection, Immunity and Inflammation, Cochin Institute, CNRS UMR 8104, Paris, France.

[2]INSERM U1016, Paris, France.

[3]Université Paris, Paris, France.

*Corresponding author: morgane.bomsel@inserm.fr

Supplemetary data

Supplementary figure 1: **HIV-1-specific-IgA and IgG are unable to mediate ADCP of HIV-1-infected CEM-NKr CCR5 CD4**$^+$**T cells by monocytes.**

CD4$^+$T cells CEM-NKr CCR5 were infected with JR-CSF clade B HIV-1 for 72hrs and incubated with 0.5µg/ml of 2F5-IgA/2F5-IgG/2G12-IgG or hIgA/IgG for 30min at 37°C. Primary monocytes were stained with 0.1µM intracellular CellTracker™ Deep Red Dye, added to opsonized infected target cells and allowed to phagocyte for 3hrs. Cells were washed, fixed, permeabilized before intracellular staining with anti-p24 FITC Ab and analyzed by flow cytometry. ADCP percentage is determined as described in the Method section. Values represent means of HIV-1-specific infected cells ADCP % ± SEM, from 3 independent experiments performed in triplicate, NS: $p > 0.05$, unpaired Student's *t*-test.

Supplementary figure 2: **Live but not apoptotic HIV-1-infected primary CD4**$^+$**T lymphocytes are phagocytosed by monocytes triggered by 2F5-IgA.**

Primary CD4$^+$T cells were infected with JR-CSF clade B HIV-1 for 72hrs. Dead cells were then removed from target cells or not before incubation with 0.05µg/ml or 0.2µg/ml of 2F5-IgA for 30min at 37°C. Primary monocytes were stained with 0.1µM intracellular CellTracker™ Deep Red Dye, added to opsonized infected target cells and allowed to phagocyte for 3hrs. Cells were washed, fixed, permeabilized before intracellular staining with anti-p24 FITC Ab and analyzed by flow cytometry. ADCP percentage is determined as described in the Method section. Values represent means of HIV-1-specific infected cells ADCP % ± SEM, from at least 2 independent experiments performed in triplicate, NS: $p > 0.05$, unpaired Student's *t*-test.

Supplementary figure 3: **2F5-IgA and 2F5-IgG do not cooperate to increase ADCP of HIV-1-infected primary CD4**$^+$**T lymphocytes by monocytes.**

Primary CD4$^+$T cells were infected with JR-CSF clade B HIV-1 for 72hrs and incubated with indicated concentrations of 2F5-IgA and/or 2F5-IgG for 30min at 37°C. Primary monocytes were stained with 0.1µM intracellular CellTracker™ Deep Red Dye, added to opsonized infected target cells and allowed to phagocyte for 3hrs. Cells were washed, fixed, permeabilized before intracellular staining with anti-p24 FITC Ab and analyzed by flow cytometry. ADCP percentage is determined as indicated in the Method section. Values represent means of HIV-1-specific infected cells ADCP % ± SEM, from at least 2 independent experiments performed in triplicate, NS: $p > 0.05$, unpaired Student's *t*-test.

Supplementary figure 4: Anti HIV-1 envelope-mediated IgG ADCP of HIV-1 infected CD4+T lymphocytes by neutrophils is inefficient.

Primary CD4+T cells were infected with HIV-1 and opsonized with 1µg of 2F5-IgA, 2F5-IgG or 2G12-IgG for 30min at 37°C. Primary neutrophils were pre-stained with 0.1µM intracellular CellTracker™ Deep Red Dye, added to opsonized infected target cells and allowed to phagocyte for indicated times. Cells were washed, fixed, permeabilized, stained intracellularly with anti-p24 FITC Ab and analyzed by flow cytometry as in Figure 4. ADCP percentage in each condition is determined as indicated in the Method section. Values represent means of HIV-1- infected cell specific ADCP % ± SEM, from at least 2 independent experiments performed in triplicate.

Supplementary figure 5: Gating strategy to quantify ADCP of HIV-1-infected cells by flow cytometry. (A) Live cells are selected based on SSC/FSC profile. **(B)** Doublets are excluded to discard $CD4^{+}T$ cells/effector cells conjugates. **(C)** Effector cells (EC) labeled with CellTracker™ Deep Red Dye (DR) are selected based on the DR fluorescence. **(D)** Finally, the percentage of EC that have phagocytosed HIV-1-infected $CD4^{+}T$ cells is determined by establishing a gate excluding the 5th percentile of negative control (in the absence of antibody) allowing the calculation of HIV-1-specific ADCP by subtracting background events, as indicated in the Method section.
